# Cattle transport network predicts endemic and epidemic foot-and-mouth disease risk on farms in Turkey

**DOI:** 10.1371/journal.pcbi.1010354

**Published:** 2022-08-19

**Authors:** José L. Herrera-Diestra, Michael Tildesley, Katriona Shea, Matthew J. Ferrari

**Affiliations:** 1 Department of Biology, The Pennsylvania State University, University Park, Pennsylvania, United States of America; 2 Department of Integrative Biology, The University of Texas at Austin, Austin, Texas, United States of America; 3 Zeeman Institute for Systems Biology and Infectious Disease Epidemiology Research, Mathematics Institute and School of Life Sciences, University of Warwick, Coventry, United Kingdom; 4 Center for Infectious Disease Dynamics, Pennsylvania State University, University Park, Pennsylvania, United States of America; Johns Hopkins University, UNITED STATES

## Abstract

The structure of contact networks affects the likelihood of disease spread at the population scale and the risk of infection at any given node. Though this has been well characterized for both theoretical and empirical networks for the spread of epidemics on completely susceptible networks, the long-term impact of network structure on risk of infection with an endemic pathogen, where nodes can be infected more than once, has been less well characterized. Here, we analyze detailed records of the transportation of cattle among farms in Turkey to characterize the global and local attributes of the directed—weighted shipments network between 2007-2012. We then study the correlations between network properties and the likelihood of infection with, or exposure to, foot-and-mouth disease (FMD) over the same time period using recorded outbreaks. The shipments network shows a complex combination of features (local and global) that have not been previously reported in other networks of shipments; i.e. small-worldness, scale-freeness, modular structure, among others. We find that nodes that were either infected or at high risk of infection with FMD (within one link from an infected farm) had disproportionately higher degree, were more central (eigenvector centrality and coreness), and were more likely to be net recipients of shipments compared to those that were always more than 2 links away from an infected farm. High in-degree (i.e. many shipments received) was the best univariate predictor of infection. Low in-coreness (i.e. peripheral nodes) was the best univariate predictor of nodes always more than 2 links away from an infected farm. These results are robust across the three different serotypes of FMD observed in Turkey and during periods of low-endemic prevalence and high-prevalence outbreaks.

## Author summary

Contact network epidemiology has been extensively used in the context of infectious diseases, primarily focusing on epidemic diseases. In this paper we use detailed recorded data about cattle exchange between farms in Turkey from 2007 to 2012, to build, analyze and characterize the directed-weighted complex network of shipments of cattle. Additionally, using outbreaks data about recorded cases of foot-and-mouth disease (FMD) in Turkey, we assess the correlation between the “farm’s” position in the network (importance) and the risk of being infected with FMD, which has been endemic in Turkey for a long time. We find some network measures that are more likely to identify high-risk and low-risk farms (in-degree and in-coreness, respectively) when proposing strategies for surveillance or containment of an infectious disease.

## Introduction

The contact structure of a population, in particular heterogeneity in the number or rate of potential contacts between individuals, is an important predictor of infectious disease transmission [1–6]. The theoretical relationship between contact structure and disease transmission dynamics has been illustrated using a variety of epidemiologically relevant contact databases [7–10]. Theory suggests that measures of contact structure are predictive of infection risk and the potential to transmit to others. Different global (eigenvector centrality, degree, coreness, betweenness) and local (random acquaintance [11]) network measures have been used to propose surveillance and vaccination strategies in static [8, 12, 13] and temporally varying [9, 14] networks. Thus, *a priori* network characterization can be used to identify candidate sites for detecting outbreaks, either in static [1, 2, 5, 15–17], temporal [18, 19], dynamic [20] or adaptive networks [21]. Moreover, these measures and their correlations are predictive of epidemic spread and can facilitate rapid targeting of interventions once an outbreak starts [22, 23]. The role of contact structure on the initial spread of infection in a naive population has been well characterized, including for the current pandemic of COVID-19 [24]. The relationship between contact structure and long-term infection risk for an endemic disease is less well characterized [25–31]. An endemic infection may experience the network topology differently from a novel outbreak [27, 32, 33], as prior infection (e.g. immunity) or interventions may alter risk and transmission of infection [34].

Livestock transportation records provide a rich resource for describing characteristics of livestock movement networks, including source location, destination, date, and batch size. Such records have been analyzed to characterize networks of interactions [35–43], and the potential consequences of network structure on the spreading of infection between farms [36, 44, 45]. However, the lack of reliable data about the infection of farms combined with detailed shipment data, has hindered our ability to build data driven models to appropriately assess the relationship between disease incidence and contact network structure in a single population, with few exceptions [46]. Instead, the impact of the interrelation between transportation network topology and disease has only been explored through theoretical simulations [13, 35, 37–40, 42, 44, 47], or through the reconstruction of who-infected-whom networks [48, 49], which describe the network of realized transmission of a specific outbreak rather than the network of potential transmission. In addition, other sources of transmission can occur through other mechanisms, including cross-border spread between premises, sharing of machinery, movement of farm workers and other forms of fomite transmission [50–54]. The development of reliable surveillance systems, both for learning about and for managing emerging or endemic diseases remains challenging. There are many unknown characteristics of transmission and control that hamper accurate decision making, including the connectivity between farms, the duration of immunity following infection, the role of multiple serotypes circulating in the livestock population and the use (and efficacy) of vaccines for spreading diseases, such as foot-and-mouth disease. Foot-and-mouth disease (FMD) is a highly contagious viral disease of cloven-hoofed species (such as cattle, 4, and pigs). In Turkey, FMD was eliminated in the Thrace region in 2010, but remains endemic in Anatolian Turkey. There are 7 immunologically distinct serotypes of FMD; two serotypes, A and O, have been endemic in Turkey continuously. A third serotype, Asia-1, has been present intermittently and re-emerged in Turkey in 2011 after going unrecorded since 2001.

Here, we use records of shipments of cattle (source, destination, date, batch size) among farms in Turkey to create an aggregated directed—weighted network. We first characterize the structure of the underlying network that may influence FMD spread. We then, analyze the occurrence of 3718 outbreaks of FMD in Turkey between 2007 and 2012 relative to the network defined by the livestock movement records. Over this time period, we compare the distribution of node-level measures for farms that were infected, farms at high risk (neighbors of infected farms) and farms at low risk (without neighbors that were infected), with any serotype or each serotype individually. Subsequently, by means of statistical models we quantify the relationship between node-level network measures and the odds of experiencing an outbreak. We compare the differences of these relationships between the endemic and epidemic periods of FMD, for all outbreaks (regardless of serotype) and for each serotype independently. We show that while all metrics were correlated with outbreak risk in the direction expected by theory, some metrics are significantly more correlated with either the occurrence, or absence, of outbreaks in both the endemic and epidemic phase.

## Materials and methods

### Shipment network

#### The data

**Fig 1:**
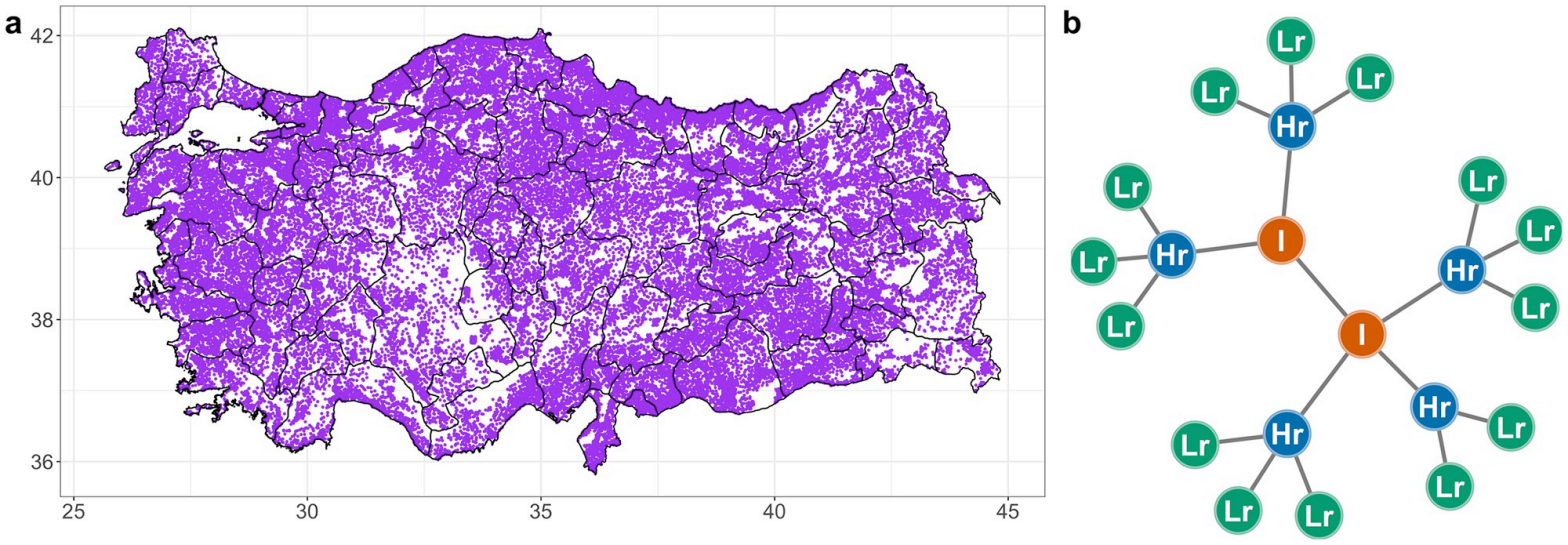
Location of epiunits in Turkey. Each purple dot represents the location of an epiunit in the map. **b.** Schematic representation for the definition infectious state of epiunits: Infected (red); high-risk (blue) and low-risk (green). Base layer of map available from https://gadm.org/download_country.html with license https://gadm.org/license.html.

The data on cattle shipments was provided by the Turkish Veterinary authorities, facilitated by the European Commission for foot-and-mouth disease (EuFMD), who granted the access to data from the TurkVet database. The highest resolution of cattle farming unit in Turkey is the holding, of which there are over 2.9 million. In each holding, birth, movement, and death data are recorded. The number of animals on these holdings can range from fewer than 5 to over 500. Since many of these holdings are small, the basic epidemiological unit for recording FMD outbreaks in Turkey is the epiunit (a village or a neighbourhood comprised of several holdings [55]); there were 49850 epiunits in Turkey (Fig 1**a**) after aggregation. The data did not identify which epiunits were markets or abattoirs, and therefore all epiunits were treated as similar. (A more detailed description in Tables A and B, Section A in S1 Appendix).

#### Network construction

We created the livestock movement (shipments) network, where each epiunit is represented by a node, and an edge is placed between two epiunits if there existed a shipment of cattle between them. In general, a graph *G* can be defined as a pair (*V*, *E*), where *V* is a set of vertices, and *E* is a set of edges between the vertices *E* ⊆ (*u*, *v*)|*u*, *v* ∈ *V*. A graph or network can be represented as an adjacency matrix *A*, defined as

$$\begin{array}{c} A=\{\begin{array}{cc} A_{ij}=w_{ij} & \text{if}\;i\;\text{is}\;\text{connected}\;\text{to}\;j\\ 0 & \text{otherwise}. \end{array} \end{array}$$ (1)

The network considered here is an aggregation of all shipments between epiunits, which created a static—aggregated network (from here on called the shipments network). The shipments network is directed (there is information about origin—destination of shipments) and weighted; the weights, *w*_*ij*_, are calculated using the frequency of unique shipments, regardless of the number of animals in the shipment, between epiunits, due to the fast-spreading nature of FMD within premises [56].

### Network description

Among the many of statistics used to characterize a complex network, we focused on those that have been used in previous studies and shown to be related to spreading dynamics; particularly epidemics [38, 42, 44, 57–65]. Further details about each of these measures in Section B in S1 Appendix).

We calculated the following global measures, which summarized properties over all nodes: density (*d*), average shortest path length (*L*); diameter (*D*); degree assortativity (*ρ*); giant strongly connected components (*GSCC*); giant weakly connected components (*GWCC*); largest eigenvalue of the adjacency matrix (λ_1_); reciprocity (*r*); the global clustering coefficient (*C*); and modularity (*Q*) using the Louvain community detection algorithm [66, 67]. We compared the global attributes of our network with an ensemble of 100 random equivalent networks as null models [68] using the Z-score of each measure. Due to the large size of the shipments network and computational limitations, the calculation of *L* and *D* were performed in the directed—unweighted version of the network (lower bound); while *C* and *Q* were calculated on the undirected—weighted version of the network which gives and upper bound [57, 69, 70].

Additionally, we calculated local (node-level) measures, which describe the characteristics of each epiunit in the network: in/out degree ($k_{i}^{in/out}$); in/out strength ($s_{i}^{in/out}$); in/out *k*-coreness ($kC_{i}^{in/out}$) and eigenvector centrality (*ec*(*i*)) (we also calculated the relative betweenness centrality (*B*_*i*_) in Section D to Section F in S1 Appendix—which did not present significant differences with the measures shown here). In each measure *i* refers to epiunits and in/out refers to the direction of the shipments used to calculate the measure.

Furthermore, for all measures with in and out modes (degree, coreness, strength), we introduced a simple measure that compares the balance between in and out shipments for each epiunit. We defined the “transmission flux” (*ϕ*) of epiunit *i* as

$$\begin{array}{c} \phi_{X\in \{degree,strength,coreness\}}^{i}=\frac{X_{in}^{i}-X_{out}^{i}}{X_{in}^{i}+X_{out}^{i}}, \end{array}$$ (2)

where *X* ∈ {degree, strength, coreness} are measures that were calculated using in and out modes. Note that $\phi_{X}^{i}\in [-1,1]$. When an epiunit *i* has $\phi_{X}^{i}=-1$, *X*_*in*_ = 0 and *X*_*out*_ ≠ 0, it is a net “source” of shipments. A value of $\phi_{X}^{i}=1$, *X*_*out*_ = 0 and *X*_*in*_ ≠ 0, identifies net “sink” epiunits. An epiunit *i* with $\phi_{X}^{i}=0$ and *X*_*in*_, *X*_*out*_ ≠ 0, interacts with its neighborhood reciprocally (*X*_*in*_ = *X*_*out*_). Our transmission flux ($\phi_{X}^{i}$) discriminates between epiunits according to their vulnerability to get infected from a spreading disease (sinks) and their ability to transmit infection (sources) [23].

Lastly, we compared our shipments network with conventional well studied networks, calculating the Spearman correlation between the matrices of correlation of node-level measures. All calculations were performed in R, and those related to complex networks statistics with the R package igraph [71].

### FMD spreading in Turkey

In addition to the data of shipments between epiunits, 6112 outbreaks of FMD (with identified serotypes) were reported to TurkVet between January 2001 and July 2012. The data included epiunits’ locations and dates of detected outbreaks. We focused our analysis to the period of time which overlapped with the shipments data (from 2007 to 2012), which included 3718 outbreaks. Our study assumes transmission due to exchange of shipments; however, transmission can occur through other mechanisms, i.e. cross-border spread between premises, sharing of machinery, movement of farm workers, and other forms of fomite transmission [50–54]. Here, these additional sources of transmission would be attributed to background risk of infection.

We first compared the distribution of local network measures for nodes that experienced outbreaks to those that did not. Then we estimated the relative effect of local measures on the odds of experiencing out outbreak. We repeated these steps for all serotypes in combination and for each serotype independently.

#### Descriptive analysis of outbreak risk

For each epiunit in the shipments network we assigned the following state labels (Fig 1b):

1. Infected epiunits (I) (red); epiunits that experienced at least one outbreak of FMD.
2. High-risk epiunits (Hr) (blue); epiunits that were directly connected (through at least one shipment) to an epiunit that experienced a FMD outbreak.
3. Low-risk epiunits (Lr) (green); epiunits that were at least at distance two (two degrees of separation) from an infected epiunit.

We then compared the distribution of local network measures of each epiunit as a function of these states, by creating several correlation planes of node-level features.

#### Statistical models of outbreak risk

To estimate the relative correlation between local network measures and outbreak risk we fitted univariate logistic regressions for each variable of interest (coreness, degree, strength, transmission flux of degree and coreness, and eigenvector centrality), in addition to a multivariate model including all these variables. We dichotomized the node states into infected nodes = 1 and all others = 0 (as defined above), to estimate the odds of infection associated with each local measure independently (univariate models) and collectively (multivariate model). Similarly we repeated these analysis with the node state dichotomized into low-risk epiunits = 1 and all others = 0 to estimate the odds of avoiding exposure associated with each local measure. Due to the high degree of skewness of the local network measures we log-transformed each of them (Fig A in Section G of S1 Appendix). We fitted these models to different temporal epochs within the time series (Table A in Section G of S1 Appendix); for each period we recalculated local network measures for the network of shipments and used the infection status during that period. Specifically, considering all outbreaks regardless of serotype, we considered the “complete” time series of all FMD outbreaks between 2007–2012; the “endemic” period, defined as before 15 February 2010 (moment when the number of outbreaks exceeded their average number for more than four consecutive days); the “epidemic” period, after 15 February 2010. Because there were many more outbreaks during the epidemic period we also considered an “epi-partial” period defined as the time period that contains all outbreaks from 15 February 2010 until the total number of outbreaks in the epi-partial period was equal than that of the total number of infected epiunits in the endemic regions (19 September 2010).

We then repeated the above for serotypes O and A independently, defining each epoch accordingly (Table A and Fig B in Section G of S1 Appendix). Using the aggregated shipments within each period defined above, we built directed-weighted networks and calculated node-level measures for each epiunit and fitted logistic regressions to the epidemic state of the node during each defined epoch. For serotype Asia-1 we considered all time points when outbreaks were present as epidemic. We classified and selected models according to the Bayesian Information Criteria (BIC).

## Results

### Characterization of the shipments network

The shipments network is a directed-weighted network which consists of *N* = 49, 580 nodes (epiunits) that are connected with *E* = 4, 746, 035 edges. The shipments network is a “complex network”; it shows a combination of features that are significantly different from random equivalent networks which are shown by the Z-scores calculated for each global measure (Table 1—Complete set of measures Table A in Section B of S1 Appendix).

**Table 1:** Network measures for the shipments network (*Calculated using the directed-unweighted version of the network. **Calculated using the undirected-weighted version of the network).

Measure	Value	Z-score
Shortest path length (*L*)	2.86*	0.01
Diameter (*D*)	20*	0.32
GSCC	97%	-
GWCC	100%	-
Largest eigenvalue (λ_1_)	3280.40	195.03
Clustering coeff. (*C*)	0.51**	23.69
Modularity(Q)	0.67**	1540.97

Specifically, the shipments network shows strong evidence of small-worldness (large clustering coefficient and small shortest path length), along with a diameter that covers the complete network in 20 steps; as well as a strong modular structure (*Q* = 0.67) (much larger that expected for a random network). There were ≈ 110 modules with more than 10 epiunits. (Table 1). The exchange of cattle occurs at many scales (Fig A in Section B of S1 Appendix) with a typical distance around 5 km and few long distance shipments (∼120 km) that connect otherwise geographically separated regions.

**Fig 2:**
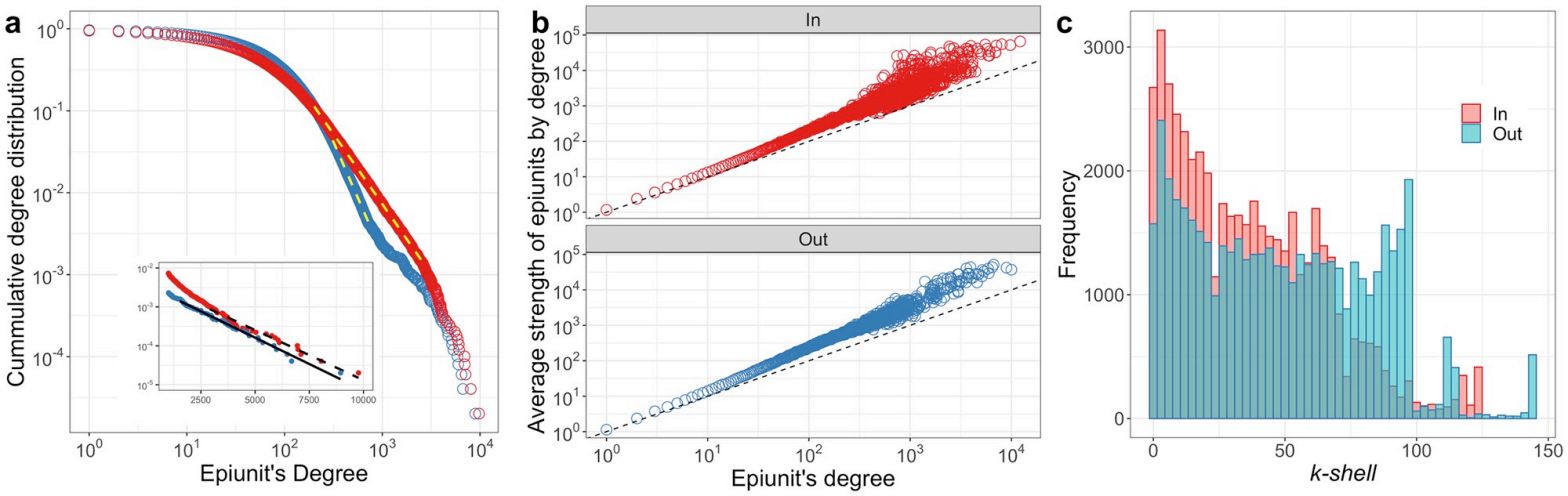
**a**. Log-Log plot of the complement of the cumulative distribution of in (red) and out (blue) degrees. Yellow dashed lines show the scale-free region of in/out degree. The inset shows the exponential decay of the tail for both distributions and the exponential fit. **b**. Average strength $\bar{s}(k)$ of epiunits with degree *k* for in/out degree/strength, as indicated in panels. Dashed line indicates *s*_*i*_ = *k*_*i*_ **c**. Frequency of epiunits in each (in—red/out—blue) *k*-shell in the network.

The degree distribution of the shipments network is strongly right-skewed. It is best-fit by a log-normal distribution (Tables A and B and Fig A in Section C of S1 Appendix), but illustrates scale-free behavior for nodes of intermediate degree (Fig 2a—yellow dashed lines) and an exponential cut-off for nodes with large degree (Fig 2a and inset) [72]. The strength of each edge (i.e. the number of shipments to (in-strength) and from (out-strength) each epiunit) is strongly correlated with degree but grows faster than degree, implying that high degree nodes also receive disproportionately more frequent shipments (Fig 2b). The nucleus [59] of the shipments network (all epiunits in the highest *k*-shell; i.e. largest coreness) is formed by 0.8% and 0.9% of all epiunits for the in and out coreness, respectively (Fig 2c). Together, the high degree of variability in node-level measures suggests that we should see highly heterogeneous outbreak risk.

Lastly, after removing all epiunits ever infected from the shipment network, along with their connections, the GSCC of the residual network is 0.96; thus, 96% of epiunits in the residual network can be reached from any other epiunit through directed connections. A full description of the features of the network and comparison to other families of network models is presented in Section D in S1 Appendix.

### FMD outbreaks in Turkey

**Fig 3:**
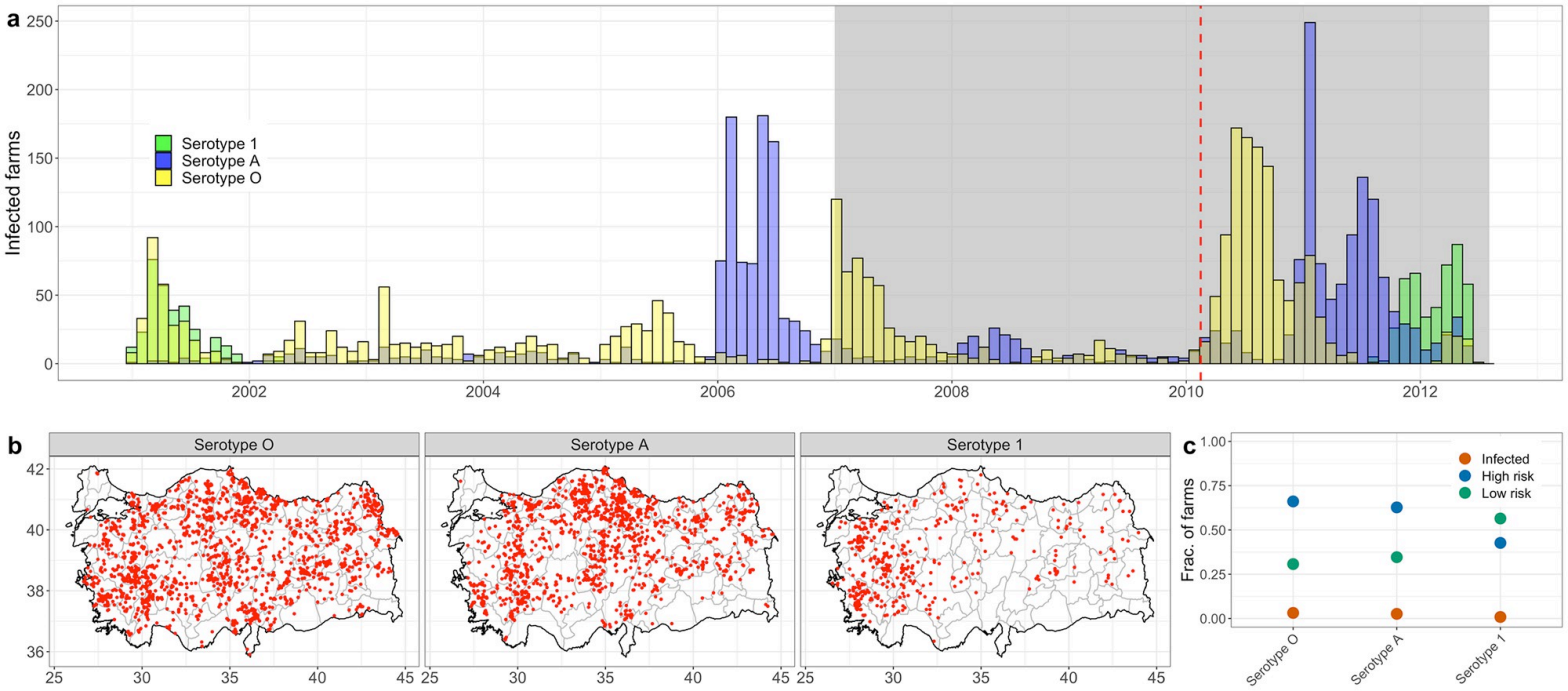
**a**. Incidence (accumulated by month) of each strain of FMD in Turkey. The gray region shows the time range where the outbreak data and the shipments data overlap (2007—2012). The red dashed vertical line delineates the endemic (left) and epidemic (right) regions, regardless of serotype, at February 15, 2010. **b**. Maps of Turkey showing the location of each of the epiunits (red points) where there occurred an outbreak of FMD. Each panel shows the location of epiunits where occurred at least one outbreak, according to serotype. **c**. Fraction of epiunits in each state (infected (red), High risk (blue) and Low risk (green), for each FMD serotype. Base layer of maps available from https://gadm.org/download_country.html with license https://gadm.org/license.html.

Serotypes O and A were present in Turkey throughout 2001–2012, with large outbreaks in 2006 and 2011 (serotype A) and 2007 and 2010 (serotype O). Additionally, there were two incursions of serotype Asia-1 in 2001 and 2012. (Fig 3a). The serotypes A and O were present across all Turkey, while the serotype Asia-1 outbreaks were disproportionately concentrated in the west of the country(Fig 3b).

Of all 49, 580 epiunits in the shipments network, 3437 were involved in at least one outbreak of any serotype between January, 2007 to July, 2012. Serotype O was detected in 1637 epiunits, 1356 epiunits were infected with serotype A, and 444 epiunits infected with serotype Asia-1. Most epiunits experienced only 1 outbreak of any serotype; though 1 epiunit had 8 outbreaks of serotype A, 1 epiunit had 5 outbreaks of serotype O, and one epiunit had 3 outbreaks of serotype Asia-1. Of all epiunits, 19 experienced outbreaks of all three serotypes.

Infected nodes were disproportionately central to the network, and low-risk nodes were disproportionately peripheral. Epiunits infected by serotypes O and A accounted for ≈ 10% of the total sum of normalized centrality (the sum of the ratio between the centrality of each epiunit and the total sum of centrality in the network; Fig A in Section E of S1 Appendix) of the shipments network regardless of the measure, despite being 3.2%, 2.6% of all nodes, respectively (Fig 3c). In contrast, epiunits infected with the Asia-1 serotype (0.8% of all epiunits; Fig 3c) comprise approximately 1% of total sum of normalized centrality. Low-risk epiunits are under-represented with respect to network centrality. For serotypes O and A, low-risk epiunits accounted for ≈ 30% of all epiunits(Fig 3c); these epiunits reflect ≈ 0.1% of eigenvector centrality and ≈ 10% out-coreness centrality, implying that these epiunits are disproportionately on the periphery of the shipments network. Epiunits that were low-risk with respect to the Asia-1 serotype were also disproportionately non-central in the shipments network, though less so than the O and A serotypes (Fig A in Section E of S1 Appendix).

#### Descriptive analysis of outbreak risk

**Fig 4:**
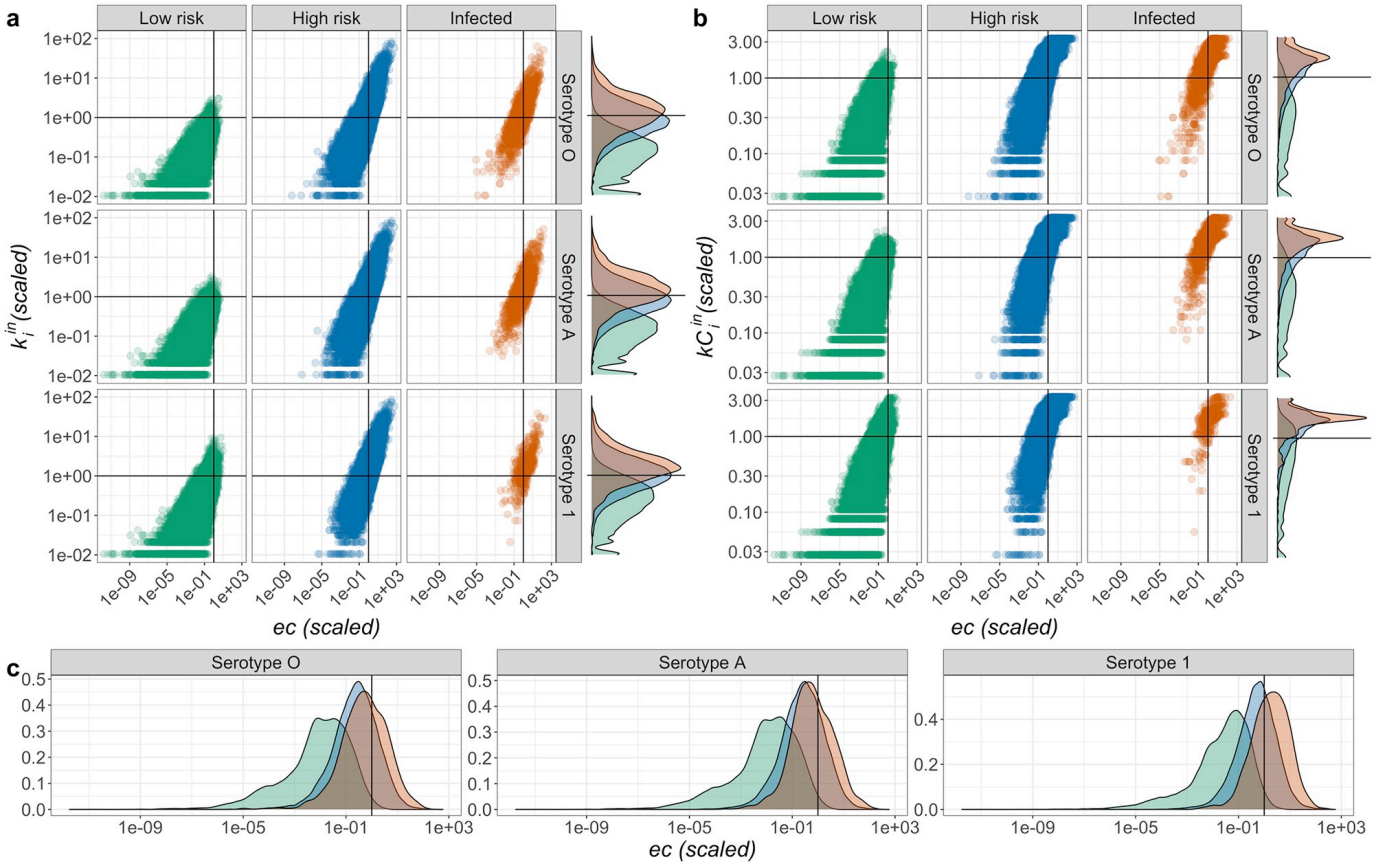
Correlation planes for different network measures of epiunits, by infectious state; i.e. eigenvector centrality (*ec*), in-degree ($k_{i}^{in}$), in-coreness ($kC_{i}^{in}$). **a**. Correlation plane (*ec*,$k_{i}^{in}$). Marginal plots correspond to densities of $k_{i}^{in}$. **b**. Correlation plane ($ec,kC_{i}^{in}$). Marginal plot corresponds to the densities of $kC_{i}^{in}$. **c**. Density plots for *ec* for each of the three serotypes. $k_{i}^{in}$, $kC_{i}^{in}$ and *ec* have been re-scaled using their corresponding mean value in the complete network. Horizontal lines show, for *k*^*in*^ and $kC_{i}^{in}$, the mean value for reference. In all panels colors correspond to low-risk (green), high-risk (blue), and infected (red) epiunits for each of the three serotypes (rows) (**a** and **b**). Vertical lines show the location of the mean value for the *ec* in all plots. Different strains of FMD are as labeled.

For all serotypes, there is a positive correlation between in-degree ($k_{i}^{in}$) and eigenvector centrality (*ec*; Fig 4a; correlation value considering all epiunits, regardless of infectious state 0.69). Low-risk epiunits had disproportionately lower in-degree ($k_{i}^{in}$) and eigenvector centrality (*ec*) than infected epiunits, with nearly all low-risk epiunits below the mean value for both measures (Fig 4a). Infected epiunits had a similar distribution of in-degree and eigenvector centrality as high-risk (neighbors of infected) epiunits for serotypes O and A (Fig 4 marginal plots). Epiunits infected with serotype Asia-1 tend to have higher eigenvector centrality than the mean for the network. In-coreness (Fig 4b) was positively correlated with eigenvector centrality. Low- and high-risk epiunits occur across the range of values of in-coreness; but, low-risk epiunits disproportionately had low values of in-coreness. Infected epiunits had disproportionately high values of in-coreness. Thus infection with all three serotypes was more likely in the nucleus of the network. The flux in degree and coreness was neither correlated with *ec* (0.12 and 0.06, respectively), nor different among node states (Fig A in Section F of S1 Appendix).

#### Statistical models of outbreak risk

**Fig 5:**
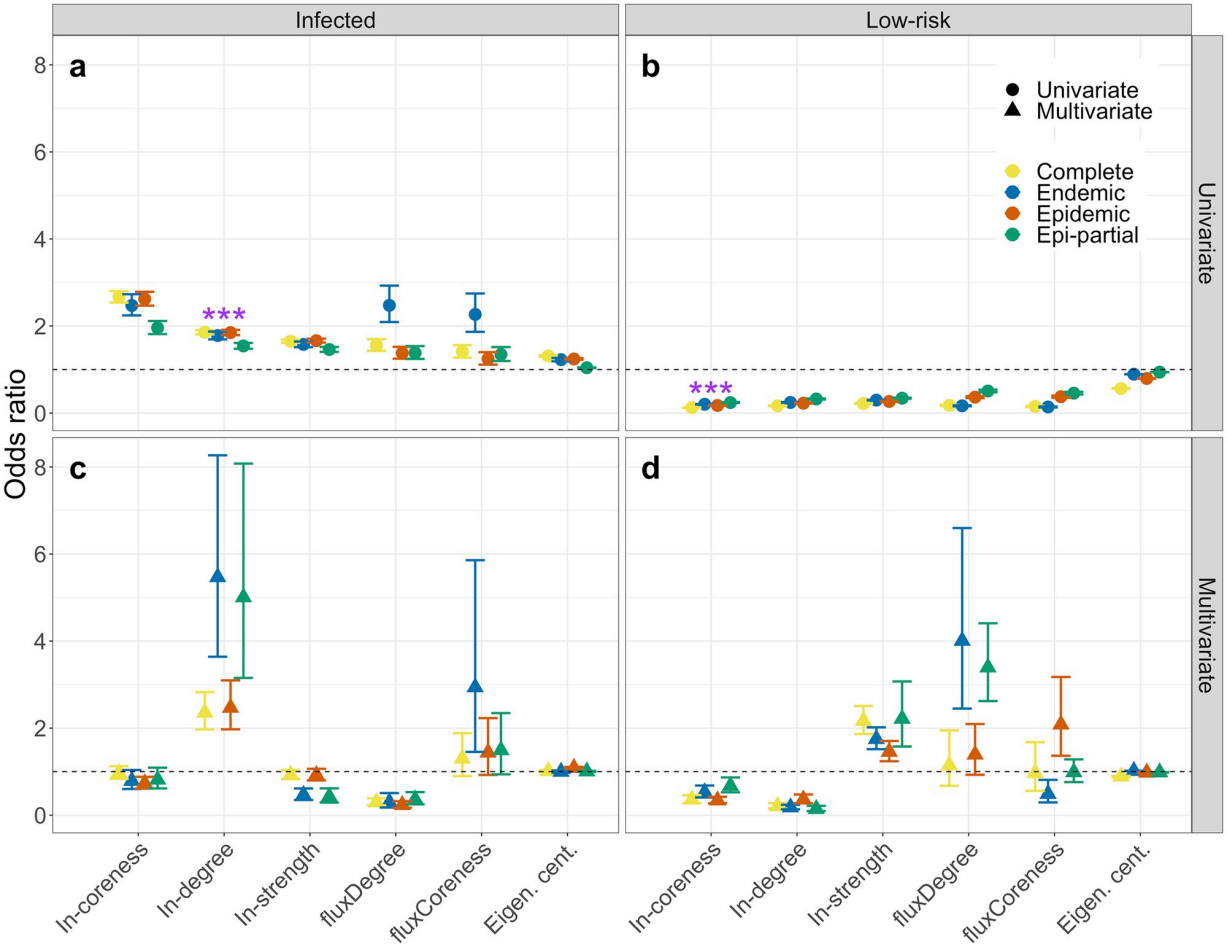
**a** and **b**) and multivariate (**c** and **d**) models. Complete network, aggregated in the corresponding period considered (2007–2012), endemic region (before February 15, 2010, epidemic region (after February 15, 2010), and epi-partial, (after February 15, 2010, until the aggregated number of outbreaks is similar to the number of outbreaks in the whole endemic region). The best univariate predictor (based on BIC) is indicated with “***” in panels (**a** and **b**). Results for each independent serotype in Section G in S1 Appendix.

The odds of being infected (with any serotype) was significantly positively correlated (*OR* > 1, Fig 5a) with all node level measures for the complete time series and in all 3 subset periods (endemic, epidemic and epi-partial). Similarly, the odds of being a low-risk node (greater than 1 edge from an infected node) was significantly negatively correlated with all measures. In-degree was the best fit among univariate models (determined by BIC) for predicting epiunits with an infected state (Fig 5a). The best predictor of low-risk state (given by lowest BIC) was the in-coreness (Fig 5b).

For a multivariate model including all variables, in-degree is the only variable that is consistently significant and positively correlated with infection of epiunits in all epochs (Fig 5c). Both in-degree and in-coreness are significant and negatively correlated with low-risk epiunits (Fig 5d). These results hold when considering independent serotypes and the different regions (Fig C in Section G of S1 Appendix).

## Discussion

Here we used a large and detailed database of cattle shipments between epiunits in Turkey, to build and characterize an aggregated directed-weighted network. Over a 6 year period from 2007–2012 the occurrence of FMD outbreaks in Turkey were correlated with node-level network characteristics. Specifically, using univariate models, in-degree (i.e. receiving many shipments) was the variable with the best fit when predicting outbreaks, while epiunits with low in-coreness (nodes on the periphery of the network) were better predicted to have low risk. These behaviors hold when considering multivariate models (in-degree is in the correct direction of association when predicting the odds of having an outbreak; and in-coreness is also still predictive of low-risk epiunits), nonetheless, the individual contribution of each variable has an unexpected behavior. Notably, these results are consistent regardless of serotype and of the different periods considered (complete, endemic, epidemic, epi-partial).

The Turkey shipments network is complex, with a combination of global measures that has not been reported for other similar networks [38, 39]. The directed nature of the network is given by the identification of origin-destination epiunits; and the weight of each edge is given by the frequency of shipments between epiunits, due to the fast-spreading nature of FMD within premises [56]. The shipments network is small-world (small shortest path length, and large clustering coefficient), also showing that in an ideal case, an infectious disease could reach a large fraction of the epiunits (*GSCC* = 97%) in a small number of generations (*D* = 20) [3].

Notably, even after removing all epiunits that experienced outbreaks and their connections, the *GSCC* of the residual network remains high (96%), a property shared among scale-free-like networks (robust under random failure), which at the same time makes them vulnerable to intentional and targeted attacks [1]. Consequently, the relatively limited penetration of FMD into the shipments network may reflect the effect of local mitigation efforts (e.g. reactive vaccination and movement controls) and structural elements of the network itself. In particular, we find that the network is highly modular *Q* = 0.67; such modularity has been shown to reduce the risk of large outbreaks [73, 74]. It is important to mention that one limitation of our data is the lack of distinction of epiunits; i.e. regular epiunits, markets or abattoirs. This may potentially underestimate transmission risk [53]; however, we are not able to estimate the scale of this underestimation. Despite this, we believe that our analysis provides strong evidence of the risk associated with transmission through the livestock network. We note that our flux measures *could* identify the importance of nodes that are strong “sinks” of shipments, such as abattoirs. Flux in degree and coreness did have large effect size for association with infection in the endemic epochs, but were not the best fit univariate measure and reversed direction. Additionally, even when other mechanisms of transmission may be in play (cross-border spread between premises, sharing of machinery, movement of farm workers, and other forms of fomite transmission) we only focused on the transmission that may occur due to the exchange of shipments [50–54].

Though FMD outbreaks between 2007–2012 were broadly distributed across the country (Fig 3b), there were distinct relationships between node-level network properties and the likelihood of an epiunit experiencing an outbreak; or, conversely that an epiunit was at low-risk for exposure to an FMD outbreak. Primarily, considering each metric independently, epiunits that recorded outbreaks (of any serotype) had disproportionately higher in-coreness and in-degree, were more likely to be net recipients of shipments, and had higher eigenvector centrality compared to epiunits that never recorded an outbreak; and the converse was true for epiunits that were greater than 1 edge away from outbreaks (Fig 4). Among all univariate models, in-degree was the best predictor of infection in the network (Fig 5a); the more shipments received by an epiunit, the more risk of infection. On the other hand, even when all univariate models predicted that low-risk epiunits would have the lowest scores regardless of the node-level measure used, in-coreness was the best predictor of epiunits that were safe from an ongoing outbreak (Fig 5b).

The univariate correlations were robust both across serotypes and during low and high prevalence periods in the time series. This observation is important as it implies a broad consistency in dynamics of exposure outbreaks. Further, analysis of risk during low-prevalence, endemic periods (e.g. 2008–2010) would give qualitatively similar assessment of the risk of outbreak (similar odds ratio) as would be expected during high-prevalence outbreaks. Similarly, analysis of any one serotype (or all serotypes combined) would give a similar assessment of risk as for any individual serotype; e.g. the re-emergence of the Asia-1 serotype in 2011 (Section G in S1 Appendix).

It is customary that strategies for surveillance and control in networks are implemented using individual network measures [8–12, 75–79]. Combining measures to better identify high-risk epiunits seems a reasonable avenue. However, we found that a multivariate model led to counter-intuitive effects. The best univariate predictors remained significant and in the expected direction in the multivariate model, but the coefficients on many of the other predictors switched direction (Fig 5c and 5d). We note that there is high correlation among the node-level measures themselves (Fig A in Section D of S1 Appendix) and their interactions with respect to outbreak risk are unlikely to be simple or linear. The intricacies of the appropriate combination of measures in a multivariarte model is an interesting avenue for further study.

Our results suggest that a plausible strategy for the placement of sensors for surveillance in the network could be applied in two sequential steps: given the complex network (1) calculate the in-coreness of the network and discard all nodes in the periphery of the network; (2) in the residual network, select an appropriate fraction *f* of the best ranked nodes according to in-degree. Furthermore, our results show that in the shipment network discarding epiunits with low in-coreness could be a potential criteria for vaccination campaigns (path to optimal allocation of resources); while the in-degree could be used to identify epiunits with high spreading impact in an ongoing outbreak (placement of sensors for surveillance).

The analysis of the aggregated shipments network opens questions which can be approached in future research. For instance, the robustness of epiunits’ importance using a temporal network approach [80]. Additionally, the possibility of implementing, proposing and evaluating current and novel strategies for control and surveillance of infectious diseases.

## Supporting information

S1 Appendix Supplemental material. File containing supplementary explanation and additional tables and figures to complement results shown.
(DOCX)
