# Supplementary material for: Cattle transport network predicts endemic and epidemic foot-and-mouth disease risk on farms in Turkey
Source: PLoS Comput Biol. 2022 Aug 19;18(8):e1010354. doi: 10.1371/journal.pcbi.1010354 (PMC9432692; doi:10.1371/journal.pcbi.1010354)
Supplement: S1 Appendix — File containing supplementary explanation and additional tables and figures to complement results shown. (DOCX) [file pcbi.1010354.s001.docx]

**Network structure and disease risk for an endemic infectious disease (Supplemental material)**

Jose L. Herrera-Diestra^1,2,*^, Michael Tildesley^3^, Katriona Shea^1,4^, and Matthew Ferrari^1,4^

^1^Department of Biology, The Pennsylvania State University, University Park, PA, USA.

^2^Department of Integrative Biology, The University of Texas at Austin, TX, USA

^3^Zeeman Institute for Systems Biology and Infectious Disease Epidemiology Research, Mathematics Institute and School of Life Sciences, University of Warwick, Coventry, UK.

^4^Center for Infectious Disease Dynamics, Pennsylvania State University, University Park, PA, USA

**Section A. Description of the data.**

*Shipment data*

Of all the data provided by Turkish Veterinary authorities, the relevant data we use in the present manuscript contains information about the date of shipment, source, and destination epiunits, along with the size (number of cattle) in each shipment (Table A). As described in the main manuscript, epiunits are assigned a unique number.

**Table A. Sample of the data provided by Turkish authorities, about source, destination epiunits and information about the shipment.**

| shipment_date | source_epiunit_id | destination_epiunit_id | shipment_size |
| --- | --- | --- | --- |
| 2007-01-01 | 21 | 38364 | 1 |
| 2007-01-01 | 28 | 45304 | 1 |
| 2007-01-01 | 73 | 9683 | 1 |
| 2007-01-01 | 123 | 23696 | 1 |
| 2007-01-01 | 131 | 42907 | 1 |
| 2007-01-01 | 215 | 13346 | 1 |
| 2007-01-01 | 254 | 31569 | 2 |
| 2007-01-01 | 313 | 17914 | 1 |
| 2007-01-01 | 373 | 31883 | 1 |
| 2007-01-01 | 389 | 2017 | 1 |

Using the data provided by the Turkish Veterinary authorities, we grouped and aggregated the data to create an edgelist containing the epiunit (node) of origin of shipment (source), the destination of shipment (destination) and the frequency (weight) of shipments between epiunits (Table B).

**Table B. Sample of the edgelist (obtained from the shipment data), used to build the directed – weighted network.**

| source | destination | weight |
| --- | --- | --- |
| 1 | 592 | 1 |
| 1 | 877 | 1 |
| 1 | 1036 | 1 |
| 1 | 1091 | 1 |
| 1 | 2247 | 6 |
| 1 | 2745 | 1 |
| 1 | 3029 | 1 |
| 1 | 3445 | 2 |
| 1 | 4183 | 1 |
| 1 | 4538 | 1 |

**Section B. Description of local and global network measures.**

***Global measures***

We calculated the following global measures in the network:

*The density,* is defined as a ratio of the number of edges to the number of possible edges in a network [5]. A value of one (complete network, where all nodes are connected to all other nodes) implies that all potential connections have been placed.

*The average shortest path length.* It represents the shortest path among two epiunits averaged over all existing pairs of epiunits in the network [6].

*The diameter* of the shipments network is the largest shortest path in the network. The bigger the diameter of a network, the less connected a network tends to be. A shorter diameter means that the number of generations for a disease to spread throughout the network is reduced.

*The degree distribution.* The degree distribution *P(k)* of the shipments network is defined to be the fraction of epiunits in the network with degree *k*. In a directed network there will be a *P(k_in_)* and *P(k_out_)* for the in and out degree, respectively.

*The strength distribution.* The strength distribution *P(s)* of the shipments network is defined to be the fraction of epiunits in the network with strength *s*. In a directed network there will be a *P(s_in_)* and *P(s_out_)* for the in and out strength, respectively.

*The degree assortativity* of the shipments network describes the tendency of an epiunit to be preferentially connected to other epiunits which have degrees similar to their own (degree assortativity close to 1). In networks with disassortative mixing, high-degree epiunits tend to be connected to epiunits with low degree and vice versa (degree assortativity close to −1) [7].

*The giant strongly/weakly connected components.* In a directed contact network, a crucial role in disease transmission is played by the strong components [8]. In the shipments network these are defined as subsets of the network where any two epiunits *i* and *j* are mutually reachable by following directed paths, and thus a disease introduced into any epiunit in a strong component can potentially reach any other epiunit in the strong component. The largest strong component is known as the giant strongly connected component (GSCC). In addition, weakly connected components (GWCC) represent sub-networks for which a path exists between any pair of epiunits, irrespective of the link direction.

*The largest eigenvalue* of the weighted adjacency matrix ***A*** of a network, denoted as *λ_1_*, is a measure of the network strength [9]. The weighted adjacency matrix ***A*** is a *N X N* matrix whose elements *a_ij_* contain the number of shipments from epiunit *i* to epiunit *j*. The largest eigenvalue of ***A*** is bounded by the average number of shipments between epiunits 〈*k*〉, as *λ_1_* ≥ 〈*k*〉, and also by *k_max_ ≥ λ_1_ ≥* max{〈*k*〉,$\sqrt{k_{max}}$} [10], where *k_max_* is the maximum number of shipments made by an epiunit to any other epiunit. As a rule of thumb, networks with higher number of links (shipments) will have a higher *λ_1_* and networks with the important nodes connected between them (known as assortative networks) will also have a higher *λ_1_* than networks where the hubs (i.e., important nodes) are not directly connected.

*Clustering coefficient*. It measures the degree of interconnection which may exist between neighbors of an epiunit of interest [1], where a high degree of clustering can reduce the size of an epidemic [11]. It is calculated as the mean of the local clustering coefficient (defined below).

**Table A. Network measures for the shipments network (*Calculated using the directed-unweighted version of the network. **Calculated using the undirected-weighted version of the network)**

**
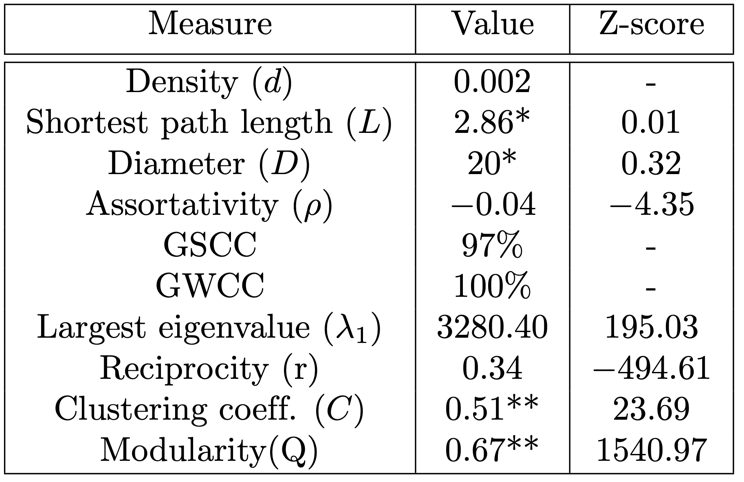
**

The shipments network shows a slight tendency to a disassortative mixing, i.e., epiunits tend to connect to other epiunits with different degrees (ρ<0); however, since ρ ~0, patterns of connectivity between epiunits resembles that of proportionate mixing (Table A). Only ~1/3 of links are reciprocal, which is a considerably smaller value than for random equivalent networks (Z-score~-500). The shipments network has a strong modular structure (Q=0.67); which is very different (higher) from the random equivalent ensemble, as shown by the large Z-score (Table A). There were n~110, where n is the number of modules with more than 10 epiunits.


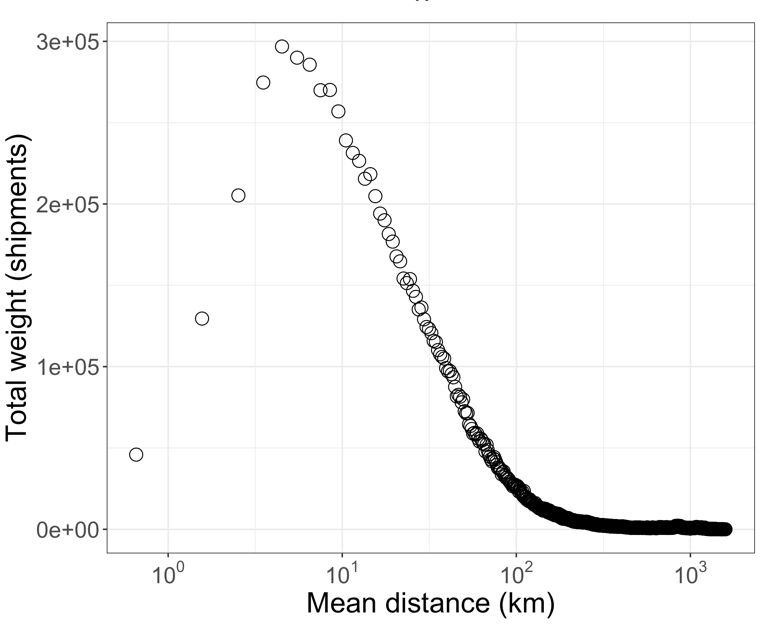


**Fig A. Total added weight of shipments versus the distance traveled for the shipment (in kilometers).**

In the shipments network, exchanges of cattle occur at many scales (**Fig A**). Above 5 km, the weight of shipments (frequency of shipments between epiunits) decreases with distance; becoming very rare for shipments longer than ~120 km. Notably, the weight and the mean distance of shipments are positively correlated for small distances, which may reflect the inherent spacing of epiunits; a restriction of interaction between close epiunits; lack of data recording at this level; among others.

***Local Measures***

Local measures quantify each epiunit’s “position” in the shipments network and their relationship to other epiunits in it. We describe local measures in terms of their absolute value, to compare one epiunit to another, and their relative value, to compare across local measures; e.g. a given epiunit is ranked among the top 10% of the distribution of degree and eigenvector centrality.

*Degree.* The degree *k_i_* of epiunit *i* in the shipments network is the number of other epiunits connected to it. In this case, the shipments network is directed and there will be two modes of degree measures: the in-degree of epiunit *i* (*k_i_^in^* - number of incident shipments of epiunit *i*) and the out-degree of epiunit *i* (*k_i_^ou^*^t^ - number of other epiunits to which epiunit *i* makes shipments to).

*Strength.* The strength *s_i_* of an epiunit *i* is the sum of the weights of the links connected to it. We use the frequency of shipments between two epiunits as their weight. In analogy to the degree, we calculate the in-strength (*s_i_^in^*) of epiunit *i* as the sum of the weights of its inward links; and the out-strength (*s_i_^out^*) as the sum of weights of the outward links.

*Local clustering coefficient.* This coefficient measures the local cohesiveness that takes into account the importance of the clustered structure on the basis of the amount of interaction actually found on local triplets. Indeed, *c_i_^w^* counts for each triplet formed in the neighborhood of epiunit *i* and the weight of the two participating edges of epiunit *i*. In this way we are considering not just the number of closed triplets in the neighborhood of an epiunit but also its total relative weight with respect to the strength of the epiunit [1].

*k-coreness,* is the largest induced subgraph of the shipments network (*G*) in which every epiunit has degree at least *k*. In general, the coreness of a vertex *v* in *G* is the largest value of *k* such that there is a *k*-core of *G* containing *v*. In the *k*-core decomposition problem, the goal is to compute the coreness of each vertex in *G*. The *k*-coreness (which will be denoted as *kC*) of epiunit *i* can be differentiated for directed networks into in-*k*-coreness (*kC_i_^in^*) and out-*k*-coreness (*kC_i_^out^*) [2]. Identifying nodes (epiunits) in the innermost core of the networks has been proven to be relevant for disease spreading [3].

*Eigenvector centrality.* The eigenvector centrality *ec(i)* of epiunit *i* is a measure of the epiunit's centrality in the shipments network that is obtained by calculating the eigenvector v_1_ associated to the largest eigenvalue *λ_1_* of the weighted adjacency matrix ***A***. The eigenvector centrality accounts for the number of all directed connections an epiunit has. Furthermore, two factors contribute to increase the eigenvector centrality of a given epiunit: (i) a higher number of direct connections to other epiunit (note that connections are weighted) and (ii) being connected to other epiunits that in turn, also have a high centrality. In this way, central eipunits in the shipments network are those that are strongly connected to other central epiunits, or epiunits with many shipments to many other epiunits.

*Relative betweenness centrality.* The betweenness centrality of an epiunit in the shipments network is defined as the frequency with which an epiunit falls between pairs of other epiunits on the geodesic (shortest) path connecting them. Consequently, the relative betweenness of an epiunit is its betweeness centrality divided by the maximum betweenness from all values in the network [4].

The relationship between ***s*** and ***k*** is super-linear (**Fig** **2b** main manuscript) (which has been observed for other networks [1,14]), where $\bar{s}\propto k^{\beta}$ ($\bar{s}$ is the average strength of epiunits by degree). For the shipments network $\beta^{in}=1.22 \pm$ 0.01 and $\beta^{in}=1.21 \pm$ 0.006 for the in and out degree, respectively. These values of $\beta$ imply that the strength of epiunits grows at a faster pace than their degree, which denotes that the more shipments an epiunit sends/receives, the more frequent the shipments tend to be sent/received by that epiunit. For the *k*-coreness of epiunits we observe that the *nucleus* [15] of the shipments network (all epiunits in the highest *k*-shell) is formed by 0.8% (0.9%) of all epiunits with respect to the in-coreness (out-coreness) of the network (**Fig** **2c** – main manuscript). There is a monotonic decrease in the number of epiunits as the *k*-shell increases using the in-coreness. However, for the out-coreness, the deepest part of the network is composed by a small number of epiunits (*k*-shell$\geq$100).

**Section C. Fitting the degree distribution.**

We fit different parametric distributions to both the in- and out-degree distribution of the shipments network using the R package univariateML [12]. From all distributions, the best fit corresponds to the log-normal distribution (lowest AIC; S4 Table). The maximum likelihood estimates of the parameters of the best-fit log-normal model are presented in Table A.

**Table A. Comparison of candidate distributions with in/out degree distribution for the shipment network.**

| **In – degree** | | | **Out - Degree** | | |
| --- | --- | --- | --- | --- | --- |
| **Distribution** | **df** | **AIC** | **Distribution** | **df** | **AIC** |
| Log-normal | 2 | 512109.7 | Log-normal | 2 | 534831.0 |
| Inverted gaussian | 2 | 517567.0 | Weibull | 2 | 535109.6 |
| Weibull | 2 | 518075.9 | Gamma | 2 | 535987.7 |
| Log-gamma | 2 | 518763.4 | Exponential | 1 | 536352.6 |
| Inverted Weibull | 2 | 520146.4 | Inverted gaussian | 2 | 544360.2 |
| Gamma | 2 | 521941.5 | Log-gamma | 2 | 545624.3 |
| Inverted gamma | 2 | 523605.9 | Inverted Weibull | 2 | 548267.9 |
| Exponential | 1 | 526564.7 | Inverted gamma | 2 | 552928.9 |
| Power law | 2 | 608202.8 | Power law | 2 | 633644.1 |
| Rayleigh | 1 | 715829.6 | Rayleigh | 1 | 667573.5 |

We plot the cumulative distributions for in-degree and out-degree of the shipments network and two representative models: log-normal distribution (best) and power law distribution (**Fig A**).


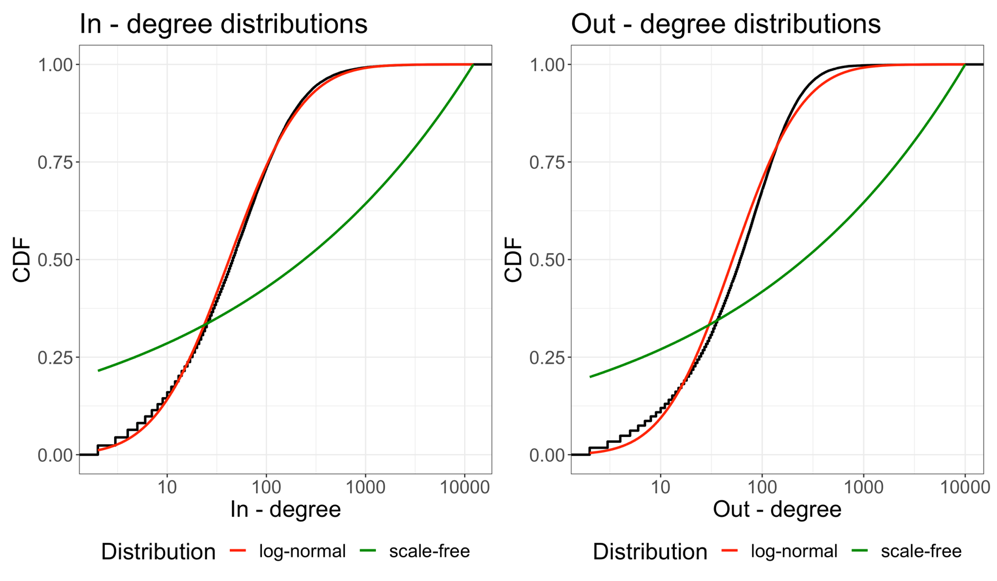


**Fig A. Cumulative distributions for the in (left), out (right) degree distributions (black). The best fit distribution (log normal – red) and a fitted scale-free distribution (green).**

**Table B. Parameter estimation.**

| **In-degree** | **Out-degree** |
| --- | --- |
| Maximum likelihood for the Lognormal model  Estimates:  meanlog sdlog  3.740792 1.340544  Data: InDegree (46958 obs.)  Support: (0, Inf)  Density: stats::dlnorm  Log-likelihood: -256052.8 | Maximum likelihood for the Lognormal model    Estimates:  meanlog sdlog  3.934222 1.240360  Data: OutDegree (48022 obs.)  Support: (0, Inf)  Density: stats::dlnorm  Log-likelihood: -267413.5 |
| Confidence intervals:     \|  \| 5% \| 95% \| \| --- \| --- \| --- \| \| meanlog \| 3.730667 \| 3.750307 \| \| sdlog \| 1.333099 \| 1.347677 \| | Confidence intervals:     \|  \| 5% \| 95% \| \| --- \| --- \| --- \| \| meanlog \| 3.925323 \| 3.943674 \| \| sdlog \| 1.234230 \| 1.246840 \| |

The degree distribution of the shipments network, when compared with several other distributions is more similar to a log-normal distribution ($\Delta_{in}^{2}=54567.3$; $\Delta_{out}^{2}=278.6$). Nonetheless, it shows a scale-free-like behavior for intermediate values of the degree ($k_{i}^{in}\in$ (200,2500) and $k_{i}^{out}\in$ (300,750), with an exponential cut-off for large values of *k* ($k_{i}^{in}>3000$; $k_{i}^{out}>1500)$ (**Fig** **2a** – main manuscript).

**Section D. Node-level correlations and comparison to alternative networks.**

We evaluate the correlations between node-level measures (**Fig A**-Left) and compare its correlation matrix with that of previously published, directed weighted networks from a variety of contexts (**Fig A**-Right). All additional weighted and directed networks were collected from <https://toreopsahl.com/datasets/.> Explanations about their sources, characteristics and references can be found in the web page.

*Neuronal:* This dataset contains the *Caenorhabditis elegans* worm’s neural network. The network contains 306 nodes that represent neurons. Two neurons are connected if at least one synapse or gap junction exist between them. The weight is the number of synapses and gap junctions.

*Intra-organizational networks:* This dataset contains four networks that are intra-organizational networks. Two are from a consulting company (46 employees – Social 1 and Social 2) and two are from a research team in a manufacturing company (77 employees – Social 3 and Social 4).

*Social 1:* Ties are differentiated on a scale from 0 to 5 in terms of frequency of information or advice requests (“Please indicate how often you have turned to this person for information or advice on work-related topics in the past three months”). 0: I Do Not Know This Person; 1: Never; 2: Seldom; 3: Sometimes; 4: Often; and 5: Very Often.

*Social 2:* Ties are differentiated in terms of the value placed on the information or advice received (“For each person in the list below, please show how strongly you agree or disagree with the following statement: In general, this person has expertise in areas that are important in the kind of work I do.”). The weights in this network is also based on a scale from 0 to 5. 0: I Do Not Know This Person; 1: Strongly Disagree; 2: Disagree; 3: Neutral; 4: Agree; and 5: Strongly Agree.

*Social 3:* Based on the employees’ awareness of each other’s knowledge and skills (“I understand this person’s knowledge and skills. This does not necessarily mean that I have these skills or am knowledgeable in these domains but that I understand what skills this person has and domains they are knowledgeable in”). The weight scale in this network is: 0: I Do Not Know This Person/I Have Never Met this Person; 1: Strongly Disagree; 2: Disagree; 3: Somewhat Disagree; 4: Somewhat Agree; 5: Agree; and 6: Strongly Agree.

*
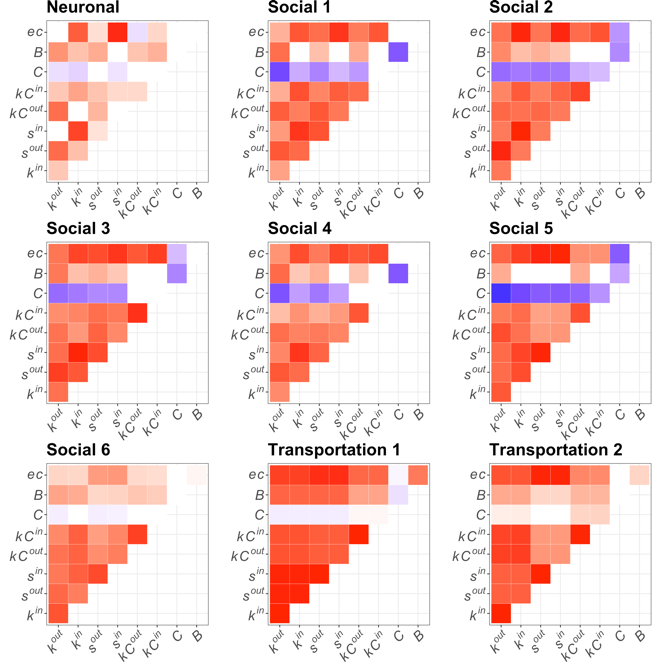

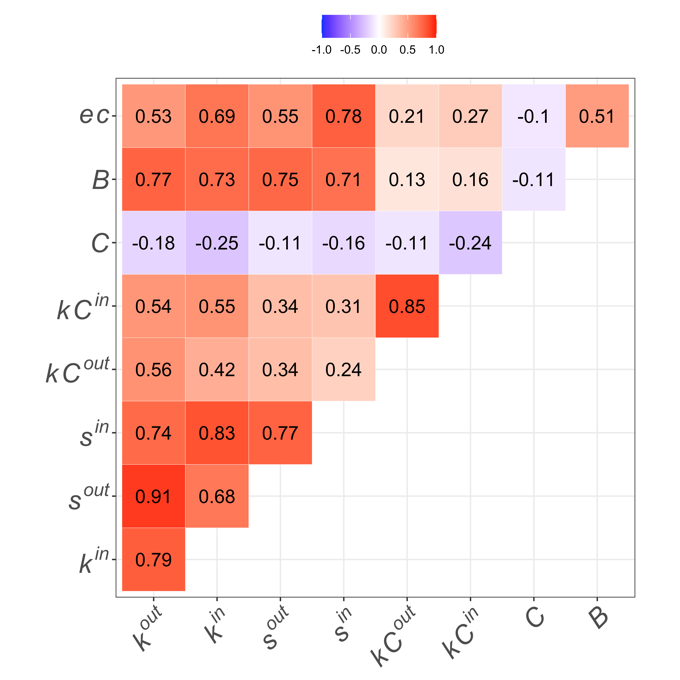
*

**Fig A. Correlation matrices between local measures. *ec*: eigenvector centrality; *B*: Betweenness centrality; *C*: clustering coefficient; *kC^in^*: in-coreness; *kC^out^*: out-coreness; *s^in^*: in-strength; *s^out^*: out-strength; *k^in^*: in-degree and *k^out^*: out-degree. Left: correlations matrix for the shipments network. Right: correlations matrices for 9 other networks from different contexts (neuronal, social and transportation) for comparison.**

*Social 4:* Ties among the researchers are differentiated in terms of advice (“Please indicate the extent to which the people listed below provide you with information you use to accomplish your work”). The weights are based on the following scale: 0: I Do Not Know This Person/I Have Never Met this Person; 1: Very Infrequently; 2: Infrequently; 3: Somewhat Infrequently; 4: Somewhat Frequently; 5: Frequently; and 6: Very Frequently.

*Social 5:* Matrix with the number of messages sent among 32 of the researchers that used an electronic communication tool (frequency matrix).

*Social 6:* The Facebook-like Social Network originates from an online community for students at University of California, Irvine. The dataset includes the users that sent or received at least one message (1,899). A total number of 59,835 online messages were set over 20,296 directed ties among these users.

*Transportation 1:* This dataset contains ties between two non-US-based airports. The weights in this network refer to the number of routes between two airports. [Airport attributes](http://opsahl.co.uk/tnet/datasets/openflights_airports.txt) are available.

*Transportation 2:* Complete US airport network in 2010. The data is downloaded from the Bureau of Transportation Statistics (BTS) Transtats site ([Table T-100; id 292](http://www.transtats.bts.gov/DL_SelectFields.asp?Table_ID=292)) with the following filters: Geography=all; Year=2010; Months=all; and columns: Passengers, Origin, Dest. Based on this table, the [airport codes](http://opsahl.co.uk/tnet/datasets/USairport_2010_codes.txt) are converted into id numbers, and the weights of duplicated ties are summed up. Also ties with a weight of 0 are removed (only cargo), and self-loops removed.

The shipments network exhibits high correlations between the in- and out- modes of measures (degree (k^in/out^), strength (s^in/out^) and coreness (kC^in/out^)) (**Fig A**-left). The betweenness centrality (B) is highly correlated with degree and strength, implying that epiunits with high movements of cattle serve as bridges that connect different regions. Clustering coefficient (C) is negatively correlated with all other measures. Eigenvector centrality (ec), a measure that evaluates the quantity and quality of connections of an epiunit, shows a positive correlation with most network measures except C; epiunits with the largest degree and strength are not the most central in the network, according to the eigenvector centrality. Epiunits with many shipments are not necessarily those that are best connected in the shipments network. Epiunits located in the nucleus of the network (deep in the network, according to in/out coreness) are not the most central in the shipments network (low correlation with ec), or bridges (low correlation with B); however, epiunits with high in-coreness tend to be epiunits with high out-coreness as well (**Fig A**-left).

To assess the general features of the shipments network and to associate it with other families of network models, we evaluate the correlations among different local measures of centrality in the shipments network (**Fig A**-left), and compare it qualitatively to other models of networks, coming from other contexts (**Fig A**-right). Qualitatively, the 5 social networks show similar negative correlation between clustering (C) and other node measures. Overall, the patterns of correlation among measures in the shipments network is most like Transportation 1 (0.91), and Social 2 (0.85) and most different from Neuronal (0.75).

**Section E. Accumulation of centrality.**

We calculate the accumulation of total network centrality as the fraction of the total centrality (sum) within each of the three classes of epiunits: infected, high risk, and low risk (**Fig A**).


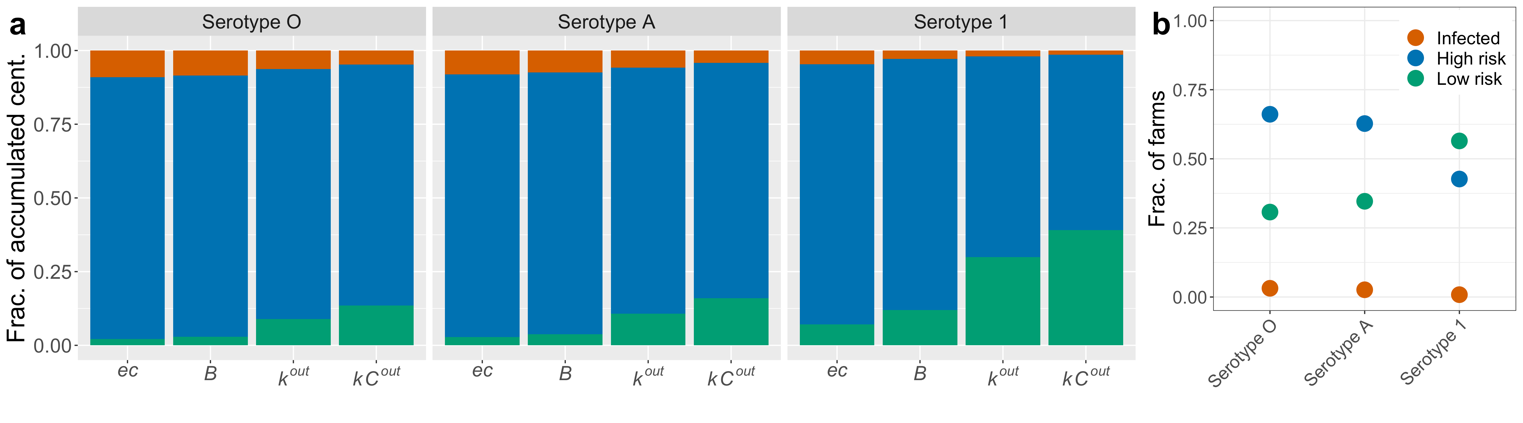
 **Fig A. (a) Fraction of accumulated centrality by epiunits for each state (infected, high risk and low risk), for different network measures: eigenvector (*ec*) centrality; betweenness centrality (B); out-degree (*k^out^* ) and out coreness (*kC^out^*). Each panel shows the results for different serotypes. (b) Fraction of farms in each state (as shown in legend) by FMD serotype.**

Regardless of serotype, infected epiunits in the network account for less than 10% of network centrality; that is, many “influential” epiunits (those with largest centrality), remained uninfected. However, for prevalent strains of FMD (O and A) these epiunits are in the “high risk” group. For serotype Asia-1, more central epiunits might still be in the “low risk” state, away from infection.

**Section F. Correlation planes.**

As in the main manuscript, we show the location of each epiunit in different correlation planes (**Fig A**).


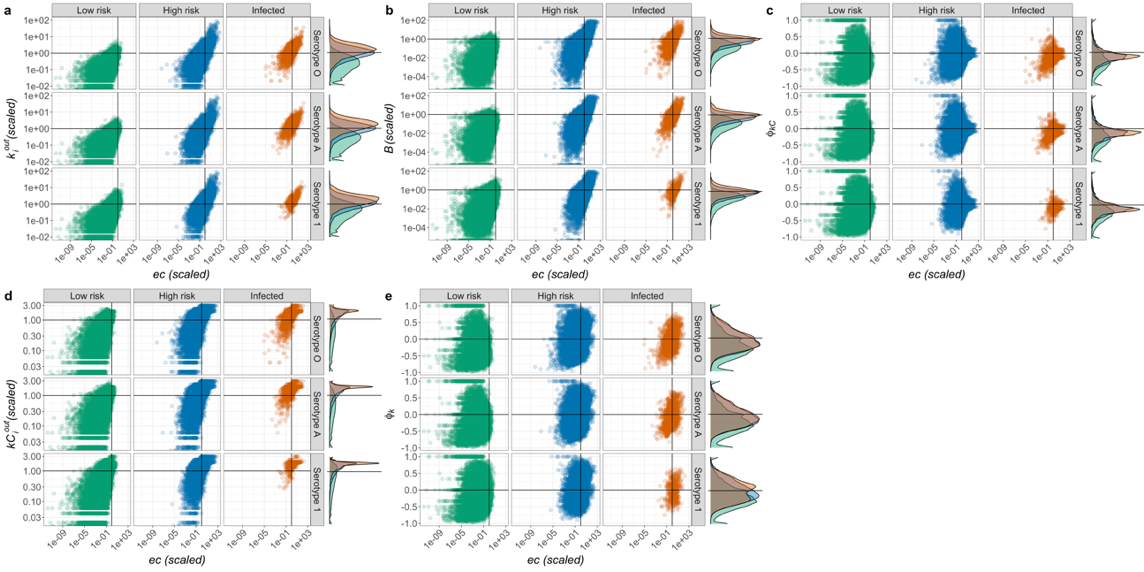


**Fig A. Correlation planes between eigenvector centrality (*ec*) and other network measures. (a) Out-degree (*k_i_^out^*); (b) betweenness centrality (B); (c) transmission flux of coreness (**$\boldsymbol{\phi}_{\boldsymbol{kC}}$**); (d) out-coreness (*kC_i_^out^*) and transmission flux of degree (**$\boldsymbol{\phi}_{\boldsymbol{k}}$**). Infectious states of epiunits in columns (Low risk, High risk and infected) and different serotypes in rows (Serotype O, A and Asia-1).**

Epiunits in the “low-risk” state are mainly located in regions of low centrality; i.e. epiunits with low rankings for any of the network measures used (**Fig A a,b,c**).

**Table A. Correlation coefficients of flux of degree and eigenvector centrality**

|  | **Low risk** | **High risk** | **Infected** |
| --- | --- | --- | --- |
| **Serotype O** | 0.00659 | 0.124 | 0.249 |
| **Serotype A** | 0.0125 | 0.127 | 0.227 |
| **Serotype 1** | 0.0866 | 0.135 | 0.197 |

**Table B. Correlation coefficients of flux of coreness and eigenvector centrality**

|  | **Low risk** | **High risk** | **Infected** |
| --- | --- | --- | --- |
| **Serotype O** | 0.00923 | 0.0637 | 0.154 |
| **Serotype A** | 0.0114 | 0.0651 | 0.175 |
| **Serotype 1** | 0.0707 | 0.0684 | 0.147 |

**Section G. Statistical model of outbreak risk**

The structure of the shipments network was such that all node-level measure was strongly skewed (**Fig A**-left). Thus, to be able to use in the statistical models, we applied a log-transformation of the variables (**Fig A**-right).


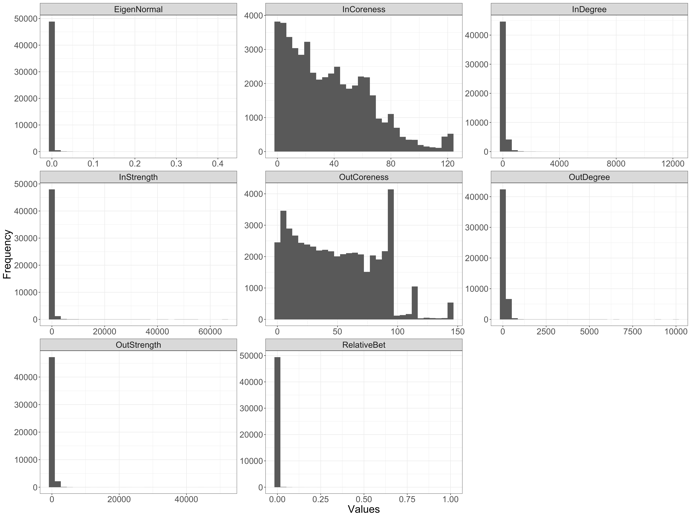

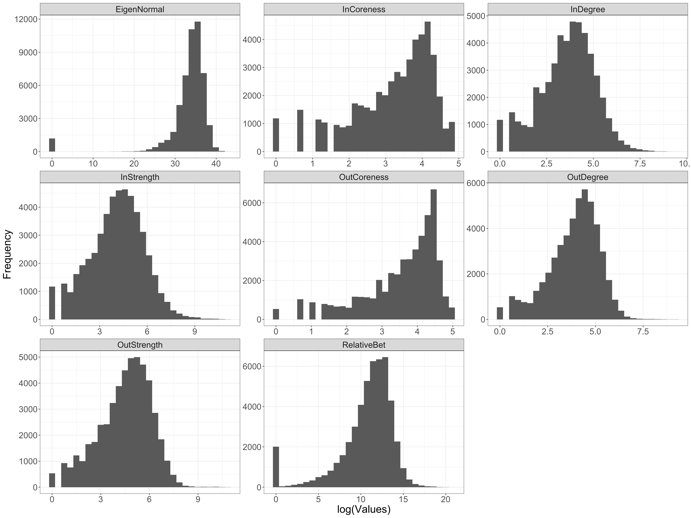
**Fig A. Left: Distributions of each network measure used in statistical models. Right: log-transformations of each measure to be used in statistical models.**

We consider 4 partitions of the outbreak time series: (1) the "complete" time series of all FMD outbreaks (regardless of serotype) between 2007-2012; (2) the "endemic", before 15 February 2010 (period of the time series where the number of outbreaks didn’t exceed the average number of outbreaks for more than four consecutive days) for outbreaks regardless of serotype; (3) "epidemic" period after 15 February 2010 (**Fig 3** – main manuscript. Vertical dashed red line). Because there were many more outbreaks during the epidemic period, we also considered an (4) "epi-partial" period defined as the time period that contains all outbreaks from 15 February 2020 until the total number of outbreaks in the epi-partial period equals that of the total number of infected epiunits in the endemic regions.

Similarly, we repeat the above partitions for serotypes O and A independently (**Fig B**). The endemic region was defined as the range before 15 February 2010 and after 15 April 2011 for serotype O (**Fig B** bottom panel, red and blue vertical lines, respectively), and before 20 October 2010 and after 15 November 2011 for serotype A, respectively (**Fig B** top panel, red and blue vertical lines, respectively). The epidemic regions were defined in the periods between 15 February 2010 - 15 April 2011 and 20 October 2010 - 15 November 2011 for serotypes O and A, respectively. Epi-partial regions were defined in the epidemic region until 28 April 2011 and 08 October 2011 for serotypes A and O, respectively. Using the aggregated shipments within each period defined above, we built directed-weighted networks and calculated node-level measures for each epiunit and fit logistic regressions to the epidemic state of the node during each defined epoch. For serotype Asia-1 we considered all time points when outbreaks were present as epidemic (**Fig B** middle panel).


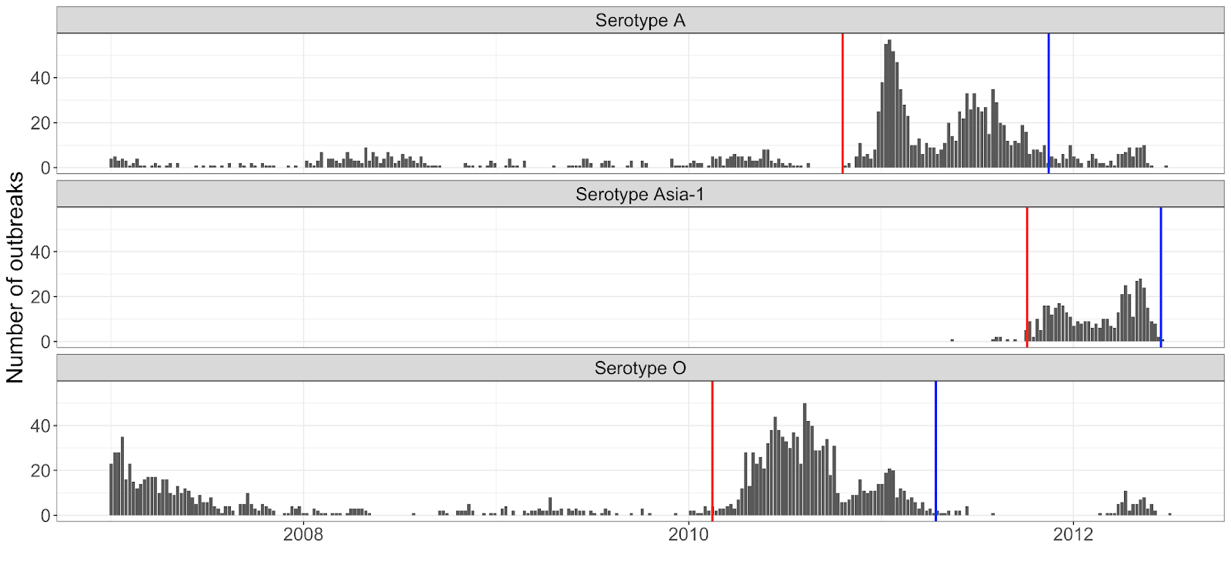


**Fig B. Epidemic (interval between the red and blue lines) and endemic (region outside the red and blue lines) for each serotype of FMD.**

We evaluated several univariate models and multivariate models to assess about the association between node-level variables and infectious state (**Fig 5** in the main manuscript, all outbreaks regardless of serotype). In most regions and for most variables, the association was the one expected; as an epiunit increases its ranking in any node-level variable, the probability of being infected increased.


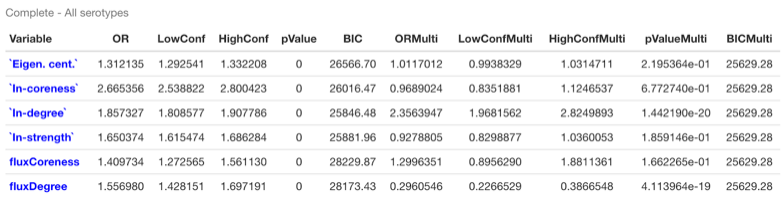

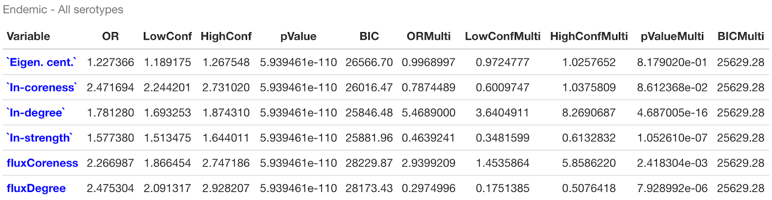

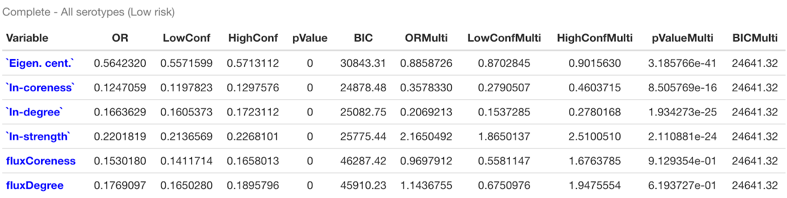

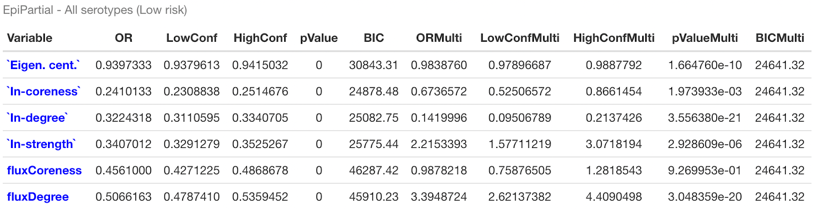

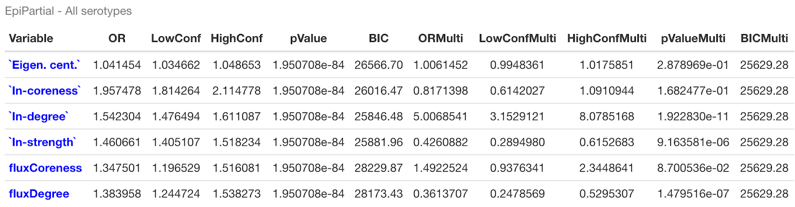

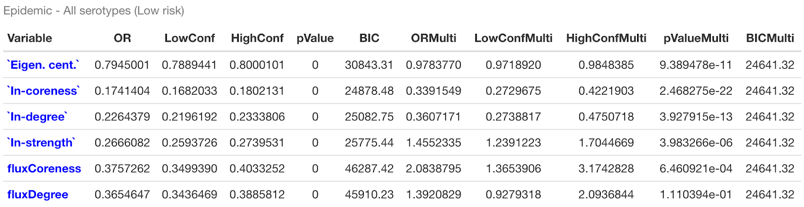

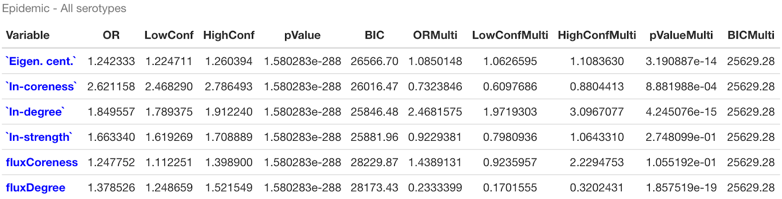

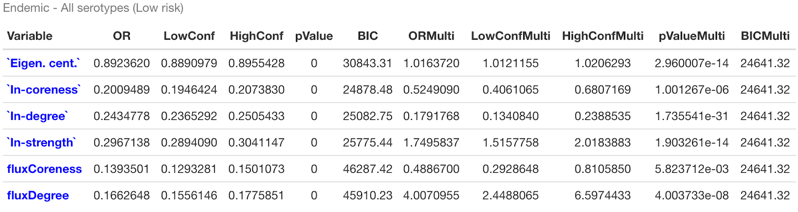


**Table A. Multivariate models for all cases (complete, endemic, epidemic and epiPartial), considering all outbreaks regardless of serotype. Evaluation of theoretical predictability of the combination of all variables associated with infection risk.**

We show in S8 Table, tables with results of the logistic regression for each period (separated by horizontal solid black lines) for all serotypes, and when the response variable was either infected epiunits (left of vertical solid black line) or low-risk (right of the vertical solid black line). Dashed blue vertical lines separate the univariate and multivariate models in the corresponding panel. Red boxes signal the variable with the best fit in the univariate models.

**Fig C** shows the same results as in **Fig 5** of the main manuscript but separated by serotype.


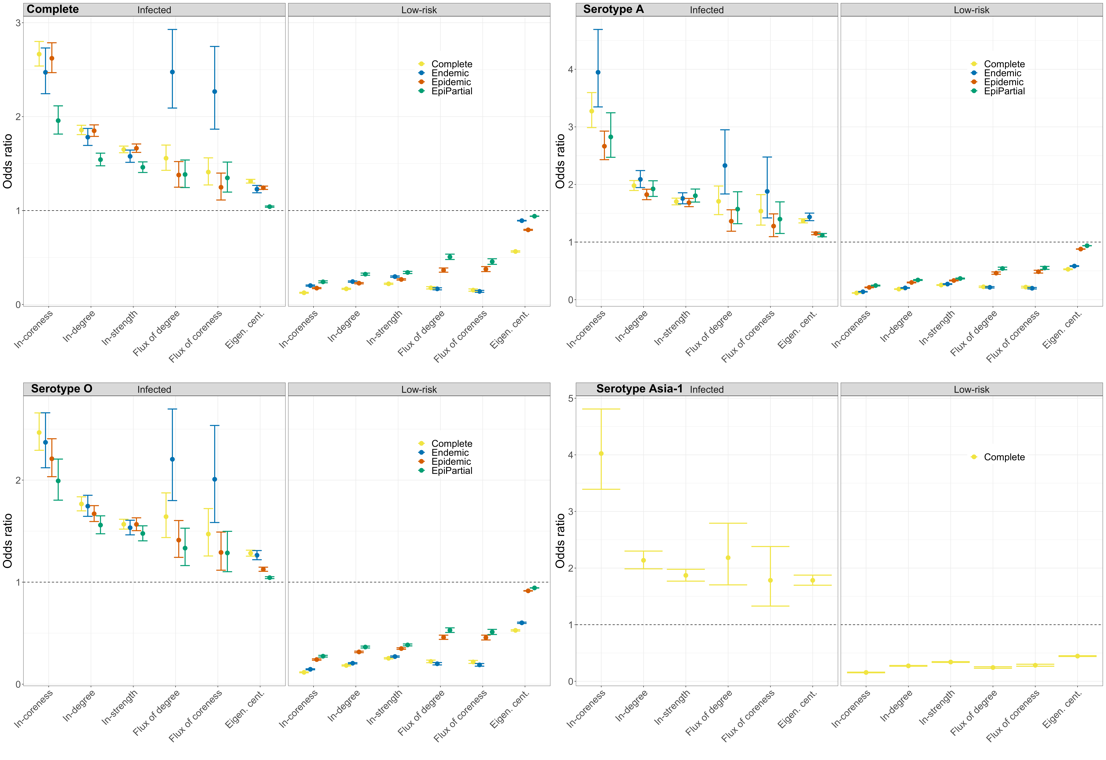


**Fig C. Univariate models for all cases (complete, endemic, epidemic and epiPartial) for each case: regardless of serotype (top), serotype A (middle) and serotype O (bottom).**

**References.**

[1] Barrat, A., Barthelemy, M., Pastor-Satorras, R. and Vespignani, A. The architecture of complex weighted networks. PNAS. 101, 11 (2004). https:// 10.1073/pnas.0400087101

[2] Batagelj, Vladimir & Zaveršnik, Matjaž. (2003). An O(m) Algorithm for Cores Decomposition of Networks. CoRR. cs.DS/0310049.

[3] Kitsak, M., Gallos, L., Havlin, S. et al. Identification of influential spreaders in complex networks. Nature Phys 6, 888–893 (2010). https://doi.org/10.1038/nphys1746

[4] Linton C. Freeman, Centrality in social networks conceptual clarification, Social Networks, Volume 1, Issue 3, 1978, Pages 215-239.

[5] Wasserman, S. and Faust, K., Social network analysis: Methods and applications, Cambridge University Press, 1994.

[6] West, D.B., Introduction to Graph Theory, Upper Saddle River, N.J.: Prentice Hall, 1996.

[7] Mark Newman, Networks: An Introduction, Oxford University Press, 2010, 1^st^. edition.

[8] Newman, M. E. J., Strogatz, S. H. and Watts, D. J., Random graphs with arbitrary degree distributions and their applications, Phys. Rev. E, 2001, 64, 026118, <https://10.1103/PhysRevE.64.026118>.

[9] Aguirre, J., Papo, D. and Buldu, J., Successful strategies for competing networks, Nature Physics, 2013, 9, 230-234. <https://doi.org/10.1038/nphys2556>

[10] Van Mieghem, P., Graph Spectra for Complex Networks, Cambridge University Press: Cambridge, 2011, 1^st^ edition.

[11] Istvan Kiss, I., Green, D. and Kao, R., The network of sheep movements within Great Britain: network properties and their implications for infectious disease spread, J. R. Soc. Interface, 2006, 3, 669-677. http://doi.org/10.1098/rsif.2006.0129

[12] Moss J (2019). “univariateML: An R package for maximum likelihood estimation of univariate densities.” *Journal of Open Source Software*, 4(44), 1863. doi: [10.21105/joss.01863](https://doi.org/10.21105/joss.01863), <https://doi.org/10.21105/joss.01863>.

[13] Rosvall, M. and Bergstrom, C. T. (2008). Maps of random walks on complex networks reveal community structure. Proceedings of the National Academy of Sciences, 105:1118–1123.

[14] Menichetti, G., Remondini, D., Panzarasa, P., Mondragón, R. J., and Bianconi, G. (2014). Weighted Multiplex Networks.PLOS ONE, 9(6):e97857, ISSN:1932-6203, <https://journals.plos.org/plosone/article?id=10.1371/journal.pone.0097857>.

[15] Carmi, S., Havlin, S., Kirkpatrick, S., Shavitt, Y., and Shir, E. (2007). A model of internet topology using k-shell decomposition. Proceedings of the National Academy of Sciences, 104(27):11150–11154, ISSN:0027-8424, <https://www.pnas.org/content/104/27/11150>.
